# Epigenetic Changes Induced by Maternal Factors during Fetal Life: Implication for Type 1 Diabetes

**DOI:** 10.3390/genes12060887

**Published:** 2021-06-08

**Authors:** Ilaria Barchetta, Jeanette Arvastsson, Luis Sarmiento, Corrado M. Cilio

**Affiliations:** 1 Department of Experimental Medicine, Sapienza University of Rome, 00161 Rome, Italy; ilaria.barchetta@uniroma1.it; 2 Immunovirology Unit, Department of Clinical Sciences, Skåne University Hospital, Lund University, 21428 Malmo, Sweden; jeanette.arvastsson@med.lu.se (J.A.); corrado.cilio@med.lu.se (C.M.C.)

**Keywords:** epigenetics, DNA methylation, autoimmune diseases, type 1 diabetes, genomic imprinting, maternal factors

## Abstract

Organ-specific autoimmune diseases, such as type 1 diabetes, are believed to result from T-cell-mediated damage of the target tissue. The immune-mediated tissue injury, in turn, is known to depend on complex interactions between genetic and environmental factors. Nevertheless, the mechanisms whereby environmental factors contribute to the pathogenesis of autoimmune diseases remain elusive and represent a major untapped target to develop novel strategies for disease prevention. Given the impact of the early environment on the developing immune system, epigenetic changes induced by maternal factors during fetal life have been linked to a likelihood of developing an autoimmune disease later in life. In humans, DNA methylation is the epigenetic mechanism most extensively investigated. This review provides an overview of the critical role of DNA methylation changes induced by prenatal maternal conditions contributing to the increased risk of immune-mediated diseases on the offspring, with a particular focus on T1D. A deeper understanding of epigenetic alterations induced by environmental stressors during fetal life may be pivotal for developing targeted prevention strategies of type 1 diabetes by modifying the maternal environment.

## 1. Introduction

**Figure 1:**
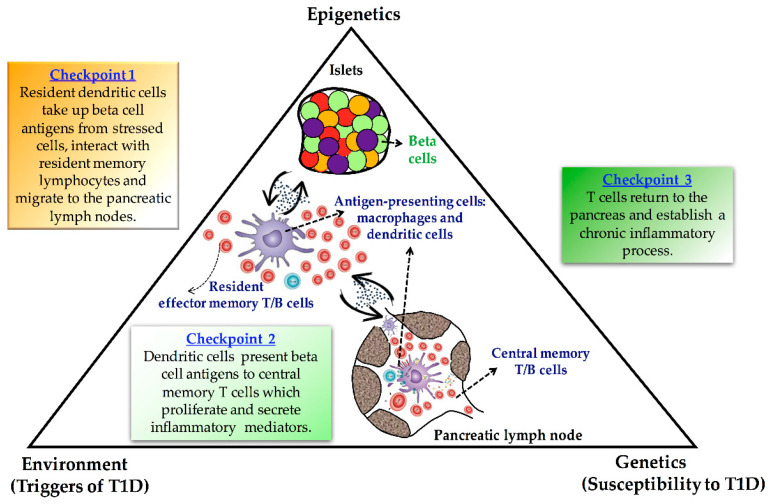
Schematic illustrating the proposed role of epigenetics as a link between genetic and environmental factors in the autoimmune destruction of the pancreatic beta cells.

Type 1 diabetes (T1D) is considered a cell-mediated autoimmune disease characterized by insulin deficiency resulting from pancreatic beta cell dysfunction [1,2]. Although the discovery of islet cell autoantibodies in 1974 shaped thinking on the pathogenesis of T1D, leading to its classification as autoimmune in nature, the etiology of the disease remains unknown. Disease-associated genes are clearly important, but numerous studies, especially those on monozygotic twins, show that heritable factors account for only 30–50% of disease susceptibility [3,4]. These findings suggest that, besides genetic contribution, environmental influences largely determine the penetrance of T1D in a genetically susceptible population. Attention in the research community has therefore focused on two major questions: (i) what are the immune mechanisms that lead to T1D, and (ii) how does interaction with environmental factors contribute to these? Addressing question (i), it is known that the immune mechanisms that lead to disease involve the generation of islet autoreactive, pro-inflammatory T cells. This process, in turn, is known to depend upon activated dendritic cells. Addressing question (ii), increasing epidemiological evidence has linked environmental agents such as diet, microbial burden, drugs, exposure to chemicals and pollutants, or country latitude with the widespread prevalence of T1D over the last decades [5,6,7,8]. Many of these environmental factors display their role in influencing disease susceptibility through changes in gene expression without altering the DNA sequence, which has been termed epigenetics [9]. Thus, epigenetic processes most probably constitute a key mechanism that bridges the gap between environmental and genetic factors in the autoimmune destruction of the pancreatic beta cells (Figure 1).

The major epigenetic mechanisms include DNA methylation, histone protein posttranslational modifications, noncoding RNA regulation, and RNA editing [10]. In mammalian species, including humans, DNA methylation is the epigenetic mechanism most extensively investigated and has a critical role in controlling gene expression [11]. Since cell type-specific DNA methylation patterns are established during embryogenesis and fetal development through a programmed process, the prenatal stages represent windows of potential vulnerability to environmental exposure-related epigenetic alterations [12]. This review briefly discusses evidence on DNA methylation alterations induced by in utero environment that may affect the risk of immune-mediated diseases on the offspring, focusing on T1D. 

## 2. Fetal Epigenetic Imprinting and Maternal Factors

Genomic imprinting refers to a parent-to-offspring transmission, where epigenetic mechanisms restrict gene expression to a single allele determined by parental origin. Thus, the control of gene expression by epigenetic inheritance confers a parent-of-origin-specific mark [13,14,15]. It has long been recognized that DNA methylation is the main mechanism responsible for establishing the imprint on one of the parental chromosomes. In humans, DNA methylation primarily occurs at cytosines in CG dinucleotides (commonly annotated as CpG, where ‘p’ represents the phosphodiester bond linking cytosine- and guanosine-containing nucleotides). Most gene promoter regions contain these CpG-rich stretches of DNA (≈500 bp), called CpG-island, and almost half of the human genes initiate transcription from CpG-islands [16]. As a consequence, methylation of promoter CpG islands is associated with transcriptional repression [17]. 

Although the diploidic state confers protection towards the consequences of genetic aberration in one gene copy during embryogenesis and fetal development, approximately 1% of the human protein-coding genome is imprinted [18]. Most of these genes are organized in clusters and expressed in the placenta [19,20]. As a direct consequence of fetal genomic imprinting, the offspring’s final phenotype is a result not only of gene sequence variation per se, but also of structural epigenetic modifications, partially obscuring any genotype–phenotype association. Hence, along with mitochondrial heritability and changes induced by in utero environment, imprinting may help explain how parent-of-origin transmission influences offspring phenotype [21]. Importantly, since this epigenetic gene-marking phenomenon occurs in germline cells, genomic imprinting modifications can be stably transmitted to several generations of cells until they are reset or lost under specific conditions [22,23].

## 3. Non-Imprinting Epigenetic Changes in Prenatal Life

Besides genomic imprinting, epigenetic modifications also occur in non-imprinted genes due to exposure to environmental factors, which exert their action predominantly by inducing different methylation profiles in CpG islets of the gene [24,25,26,27]. Like genomic imprinting, non-imprinting-related epigenetic changes are stable and heritable across generations of cells and organisms [28,29,30]. Therefore, non-imprinting epigenetic changes can be viewed as a functionally adaptive rearrangement of gene expression under environmental pressure. Fetal exposures to environmental and maternal factors may induce permanent physiological changes, termed “programming”, potentially leading to a variety of diseases later in life [31,32,33,34,35]. Indeed, both animal [36,37,38] and human [39,40,41] studies have indicated that environmental exposure experienced in utero may determine offspring phenotypic outcomes through epigenetic modulation of gene transcription. For example, it has been shown that maternal diet and nutrition patterns early in life predispose to increased cardiovascular risk, metabolic disorders, and immune impairment [42,43,44]. Moreover, insufficient intake of fruits and vegetables and high consumption of modern processed foods during pregnancy have been associated with systemic low-grade systematic inflammation [45,46]. Such maternal inflammation is believed to pass an inflammatory ‘code’ through epigenetic modifications to the offspring and influence the programming of the offspring’s immune system [47]. 

Although the existence of a “legacy” leading to permanent effects of in utero and early-life environmental exposures on unfavorable outcomes later in life has been demonstrated in prospective studies, only recently has it been recognized that these effects are mediated through epigenetic mechanisms. Thus, a genotype–phenotype mismatch could be partially attributable to external pressures that can reprogram the expression of genes related to immunity and metabolism, thereby leading to a pathological phenotype. This fits with data showing that, in mice, a maternal diet supplemented with methyl donors enhanced the severity of allergic airway disease inherited transgenerationally [48]. Therefore, changes in the DNA methylation pattern of target genes during the embryonic period could modify allergic airway disease’s heritable risk. Additionally, there is evidence in humans that site-specific changes in epigenetic marking at the Retinoid-X Receptor alpha (RXRA) promoter region in umbilical cord blood cells are negatively associated with maternal carbohydrate intake during early pregnancy. Remarkably, these epigenetic modifications correlated with childhood adiposity later in life [49]. Together, these studies support a link between non-imprinted epigenetics in fetal development and phenotypic changes in offspring.

## 4. Epigenetic Changes of Immune-Related Genes

A growing number of studies highlight the importance of epigenetic mechanisms in hematopoietic lineage choice [50], antigen-receptor rearrangement [51], allelic exclusion [52], and immune responses to pathogens [53,54]. Along with controlling T cell central tolerance in the thymus by processes related to methylated histones and miRNAs, epigenetic mechanisms also regulate peripheral tolerance. For example, it has been shown that the activity of Foxp3 protein, the master regulator for Treg cell development and immunosuppressive function, is regulated post-translationally by acetylation and deacetylation [55,56,57]. Indeed, alterations in this process lead to insufficiency in natural Tregs and impaired development and function of inducible Tregs [58,59]. In support of this notion, a study in mice demonstrated that treatment with the DNA methylation inhibitor 5-azacytidine causes experimentally induced autoimmune arthritis [60]. Studies in monozygotic twins discordant for psoriasis have also shown that changes in DNA methylation between unaffected and affected twins correlated with changes in the expression of genes involved in the immune response [61]. Finally, one study found that monozygotic twins discordant for T1D exhibit significant differences in methylation patterns in CD14^+^ monocytes [62].

Epidemiological studies investigating the effects of maternal stress on offspring have shown that prenatal exposure to maternal adverse life events results in lasting and broad functional DNA methylation changes in innate and adaptive immune genes and genes involved in glucose metabolism. In particular, the objective prenatal maternal stress experienced during the 1998 Quebec Ice Storm directly correlated with a specific DNA methylation pattern in CD3+ T cells, saliva, and whole peripheral blood of offspring, almost thirteen years after birth [63]. Interestingly, the long-lasting impact of traumatic stress on the methylation pattern of CpG sites was even detected in several genes involved in both T1D [63] and type 2 diabetes (T2D) pathways [63,64].

Given these findings, it appears plausible that fetal epigenetic changes triggered during the prenatal environment may induce long-lasting effects on offspring outcomes in later life. However, the lack of fetal cord blood cells did not allow these authors to demonstrate whether a stress-induced DNA methylation profile may already occur in the prenatal period or early in life. 

## 5. Fetal Epigenetic Changes: Studies on Cord Blood Cells

Different conditions may influence the fetal immune system’s development during pregnancy and, consequently, the risk of immune-related diseases. For example, maternal obesity (body mass index (BMI) ≥ 30 kg/m^2^) has been associated with several alterations in the perinatal immune system. In particular, maternal BMI during pre-pregnancy or early gestation affects DNA methylation in the offspring’s peripheral blood cells [65,66]. In accordance, Sureshchandra et al. [67] revealed that maternal pre-pregnancy BMI correlates inversely with overall methylation levels in cord blood samples. Interestingly, the most significant methylation changes occurred within genes associated with cancer (WNT16) and diabetes (BTN3AI). An important study by Wilson et al. [68] showed a reduction of eosinophils and CD4+ T helper cells, reduced monocytes and dendritic cell responses to Toll-like receptor ligands, as wells as increased plasma IFN-γ and IL-6 levels in cord blood cells of newborns from obese in comparison with those from lean mothers.

Other evidence supporting that maternal lifestyle and environmental exposures can influence the epigenetic programming of the offspring’s immune system is provided by Nemoda et al. [69], who found that maternal depression affects T cell DNA methylation profiles in the offspring. However, the authors did not see significant DNA methylation changes associated with depression in T lymphocytes from the antepartum maternal samples. Therefore, changes in the prenatal environment induced by maternal depression may exert long-lasting effects on immune functions in the periphery and the central nervous system of the offspring.

Other researchers have found that in-utero exposures to environmental factors, such as cigarette smoking during pregnancy [70,71,72], maternal diet [73,74,75,76], and microbial exposures [77,78], also have a dramatic influence on the risk of allergic disease in the offspring by altering fetal lung development and immune function [79]. Results from “The Managing Asthma in Pregnancy (MAP) Study” provided the first demonstration that exposure to maternal asthma during pregnancy is associated with alterations in the DNA methylation profile of infants’ peripheral blood. Among the sixty-eight genes differentially methylated, key regulatory pathways concerning developmental, metabolic, and inflammatory processes were most involved [80]. Interestingly, prenatal exposure to maternal cigarette smoke has been linked to the abnormal DNA methylation status of the 5′-CpG-island in the thymic stromal lymphopoietin (TSLP [81]), a key immune cytokine gene involved in the pathogenesis of asthma [82,83], atopic dermatitis [84], and pediatric eosinophilic esophagitis [85].

Taken together, these studies illustrate that epigenetic changes induced by prenatal maternal conditions such as maternal obesity, maternal depression, or cigarette smoking during pregnancy confer an increased risk of immune-mediated diseases in the offspring. In this regard, a particular focus should be given to the study of maternal lifestyle factors in the development of autoimmune diseases, which are largely prevalent among women of reproductive age. 

## 6. Epigenetics in T1D: The Missing Piece of the Puzzle

In T1D, insulin-producing beta cells are destroyed by autoimmune mechanisms, resulting in insulin-deficiency and hyperglycemia [1,2]. Although it is believed that genetic and environmental factors play critical roles in T1D development, a long-term puzzle in the diabetes field has been how autoreactive T cells mistakenly destroy beta cells. Thus, dissecting the epigenetic architecture at the crossroads between genes and the environment could reveal the missing piece of the T1D puzzle (Figure 1).

### 6.1. Genetics

Over the past thirty years, extensive population studies have provided an explanation for nearly 80% of the heritability of T1D [86,87]. The strongest genetic risk factor for T1D is attributable to the Human Leukocyte Antigen (HLA) class II alleles, which account for up to 50% of genetic T1D risk [88,89,90,91]. Outside of the class II region, the strongest susceptibility is conferred by HLA class I allele B*39 [92]. Among non-HLA genes, some loci weakly contribute to disease onset, such as the insulin gene (INS), tyrosine phosphatase non-receptor type 22 (PTPN22), cytotoxic T-lymphocyte-associated protein 4 (CTLA4), interleukin 2 receptor α (IL2RA), C-type lectin domain containing 16A (CLEC16A), cathepsin H (CTSH), interferon-induced with helicase C domain 1 (IFIH1), CAPSL-IL7R, Th1 transcription factor STAT4, tyrosine phosphatase non-receptor type 2 (PTPN2), and others [93,94,95,96].

### 6.2. Genome Imprinting

In addition to the predisposing genes identified, the effect of a small number of T1D-associated genes may be mediated through imprinting. It is thus conceivable that impaired fetal imprinting can lead to T1D development in several conditions. Indeed, genetic imprinting on chromosome region 6q24 PLAGL1-HYMAI is associated with transient neonatal diabetes, a rare form of diabetes whereby an increased dosage at the chromosome 6q24 region leads to impaired glucose regulation and diabetes. Notably, near half of the cases of neonatal diabetes have the condition for life [97,98,99,100,101,102]. Moreover, impaired genomic imprinting seems to influence the development of polygenic T1D [103] and T2D [104]. 

### 6.3. Non-Imprinting Epigenetic Changes

Despite evidence linking genetics with disease T1D susceptibility, they are not likely the primary driver. It should be noted that T1D incidence has increased worldwide over the last few decades at an average of ~3% to 5% per year [105], which is too rapid to be explained only by enhanced genetic disease susceptibility in the background population. If this trend continues, T1D incidence will double in the next 20 years. Interest has therefore focused on environmental factors that might trigger and/or accelerate the disease. The role of environmental factors in T1D development is also supported by a plethora of findings demonstrating that the concordance rate in monozygotic twins for T1D ranges from 13% to 60% according to the age at disease onset, insulin genotype, and latitude [106,107,108,109,110,111]. Given the evident importance of the overriding environmental influence on T1D development, it appears plausible that environmental epigenetic modifications during prenatal development may be one of the factors that are associated with an increased risk for developing T1D [112]. In particular, exposure to an adverse in utero milieu may induce epigenetic effects on DNA, permanently modifying the expression of immune genes and islet cell function-related genes. Perhaps the most compelling evidence to date on the influence of the intrauterine environment on T1D risk comes from a “migration” study performed in Sweden, a country with the second-highest level of T1D in the world. This study demonstrated that being born in Sweden increases the risk for T1D even in children with an origin in low-incidence countries, whereas T1D risk did not vary in children immigrating to Sweden at an early age for adoption and immediately introduced into Swedish families [113]. In line with this observation, data from the Skåne area in the southern part of Sweden suggested that high exposure to air pollution (i.e., nitrogen oxides and ozone) during pregnancy represents a risk factor of developing T1D in offspring [114]. Indeed, evidence exists that nitrogen oxides act as an epigenetic regulator of gene expression by controlling histone posttranslational modifications [115]. Moreover, a study demonstrated that disruption of miRNA expression profiles by ozone inhalation is associated with inflammatory and immune response signaling [116]. Consistent with this, epidemiological studies have shown that children exposed to smoking during fetal life are at higher risk of developing T1D in childhood [117]. 

Decades of research have provided evidence suggesting that certain viruses, especially human enterovirus, are putative environment-derived disease modifiers in T1D [118,119,120]. Remarkably, maternal enteroviral infection during pregnancy has been considered a risk factor for T1D onset during childhood and adolescence in several studies [121,122,123,124,125,126,127,128,129,130,131,132,133,134]. In keeping with the crucial role of epigenetic modification in early development, it is tempting to speculate that maternal viral infection during pregnancy can give rise to stable changes in immune-related genes by epigenetic mechanisms. This is an attractive idea because, if confirmed, the infection-induced epigenetic modification could contribute significantly to the offspring’s risk of T1D later in life. In support of this notion, recent studies have shown that enterovirus can alter miRNA-directed suppression of pro-inflammatory factors within pancreatic beta cells [135] and pancreatic ductal-like cells [136,137]. Likewise, Rhinovirus (another important member of the *Picornaviridae* family, as human enteroviruses) affects both the methylation status and the expression of pro-inflammatory cytokines in epithelial cells [138]. Hence, non-imprinting epigenetic modifications induced by maternal viral infections may represent one mechanism through which viruses contribute to T1D.

### 6.4. DNA Methylation Signature in T1D

Although the non-structural genetic component of T1D susceptibility remains to be determined, remarkable progress has been made in elucidating the epigenetics of T1D. As with other autoimmune diseases, DNA methylation has been the most extensively studied epigenetic signature in T1D. The major studies are shown in Table 1.

Studies in monozygotic twins have been critical to strengthening the hypothesis that DNA methylation is involved in T1D etiology. A genome-wide DNA methylation analysis of monocytes from monozygotic twins discordant for T1D conducted by Rakyan and colleagues [62] revealed the presence of T1D-specific methylation variable positions (T1D-MVP) in the diabetic co-twins. They found that the epigenetic changes in autoantibodies-positive individuals occurred before the diagnosis of T1D, which excludes the possibility of an association between methylation profile and post-disease dysmetabolic environment. Remarkably, T1D-MVP-associated genes included several genes known to be associated with T1D or immune responses, such as HLA class II, HLA-DQB1, Regulatory Factor X-Associated Protein (RFXAP), Nuclear Factor Kappa B Subunit 1 (NFKB1A), Tumor Necrosis Factor (TNF), and Glutamate Decarboxylase 2 (GAD2). Of note, the GAD2 gene encodes the islet cell-specific (65 kDa) form of glutamic acid decarboxylase (GAD65), which is one of the major autoantigens in T1D [150]. Moreover, an epigenome-wide association study in 52 monozygotic twin pairs discordant for T1D in three immune effector cell types (that is, CD4^+^ T cells, CD19^+^ B cells, and CD14^+^CD16^−^ monocytes) showed significant enrichment of differentially variable CpG positions in T1D twins when compared with their healthy co-twins and healthy controls [144]. It is also important to note that non-twin studies using T1D patients and healthy individuals have demonstrated differences in methylation profiles between T1D patients and controls [141,151]. Indeed, recent research has shown that DNA methylation is involved in regulating the genetic and environmental influence of T1D at the CTSH locus [149]. 

### 6.5. Maternal Autoantibodies and Their Role in T1D

Although the relationship between maternal antibody transmission and antibody-mediated diseases such as systemic lupus erythematosus [152,153] and thyroiditis [154,155] is widely recognized, the pathogenic role for maternal autoantibodies in T cell-mediated autoimmune diseases remains controversial. Part of this uncertainty is due to studies in nonobese diabetic (NOD) mice, a well-known animal model for T1D, showing that maternal transmission of beta cell-specific autoantibodies is necessary for inducing [156] or accelerating [157] the disease development. In contrast, more recent studies provide evidence that fetal exposure to insulin autoantibodies (IAA) did not increase the risk of diabetes development in NOD mice [158]. In humans, epidemiological data are also contradictory. Some studies have reported an increased frequency of beta cell-specific autoantibodies in cord blood of children who developed T1D, suggesting that this might represent a possible risk factor [159,160]. In contrast, the German BABYDIAB Study has demonstrated that offspring born to mothers with T1D who were positive for autoantibodies against islet-specific autoantigens linked to T1D (namely GAD65 and/or tyrosine phosphatase-related islet antigen 2 tyrosine phosphatase-related islet antigen 2, IA-2) at birth were at lower risk of T1D than offspring who were autoantibody-negative. Notably, the risk remained reduced after adjustment for potential independent confounders, such as maternal diabetes duration, birth weight, and gestational age [161], suggesting a protective role of fetal exposure to islet autoantibodies against T1D in offspring. In support of this hypothesis, accumulating data from epidemiological studies have revealed that the risk of developing T1D is low in infants born to mothers with T1D [162,163,164,165,166,167].

In the context of the Diabetes Prediction Study in Skåne (DiPiS), we studied the inflammatory, autoantibodies, and lymphocyte profiles in cord blood cells of children born to mothers either with T1D, gestational diabetes, or healthy mothers [168]. Interestingly, cord blood from children born to mothers with T1D showed increased IL-1β, IL-8, and TNFα levels and a higher frequency of CD4+ CD25+ T cells. Particularly, the CD4+ CD25+ T cells’ increase correlated with the anti-GAD65 antibodies’ titer. Remarkably, early modifications of inflammatory and immune patterns were absent in children born to mothers with gestational diabetes and without the islets’ autoantibody [168]. These results rule out the possibility that early changes in the immune system may have been induced by other factors linked to maternal diabetes, such as hyperglycemia. Overall, these data suggest that fetal/early-in-life epigenetic mechanisms might be involved in the susceptibility to islets’ autoimmunity and T1D.

## 7. Conclusions

The studies outlined here provide converging evidence to suggest that maternal factors are associated with increased risk for developing autoimmune diseases, such as T1D, through epigenetic changes in fetal life. However, there remains skepticism about whether in utero exposure to environmental factors may modify the immune profile and, subsequently, the risk of T1D later in life through epigenetic modifications. Therefore, additional birth cohort studies with long-term follow-up are needed to gain a more comprehensive understanding of how environmental cues during intrauterine life modulate the developing immune system. The use of Guthrie cards, state-of-the-art automated platforms for high-throughput epigenomics**,** and single-cell genomics in cord blood samples in established prospective cohorts hold promise to facilitate our understanding of gene–environment interaction in early life [169]. Identification of epigenetic modifications induced by prenatal environmental exposures associated with a higher risk of autoimmune diseases and T1D later in life will be of utmost importance, as this may provide better for disease prevention strategies already in utero.

## Figures and Tables

**Table 1:** Studies on DNA methylation and type 1 diabetes.

Reference/Year	Method	Sample	Results
[139]/2010	Genome-wide DNA methylation	Whole blood	Association of 19 CpG sites with risk of diabetic nephropathy
[62]/2011	Epigenome-wide association study (EWAS)	Monocytes	Presence of T1D-specific methylation variable positions in the T1D-affected co-twins
[140]/2012	Methylation of specific genes	Whole blood	Association of CpG methylation at the INS locus with T1D
[141]/2013	Methylation of specific genes	Peripheral blood	Effect of IL2RA risk alleles on T1D may be partially mediated through CpG methylation change
[142]/2014	Methylation of specific genes	Peripheral blood	Decreased IGFBP1 DNA methylation levels are associated with T1D
[143]/2015	Genome-wide DNA methylation	Whole blood	Subjects with T1D and proliferative diabetic retinopathy exhibit altered DNA methylation patterns in blood
[144]/2016	Epigenome-wide association study	T cells B cells Monocytes	T1D-associated differentially variable CpG positions are located in genes involved in immune cell metabolism
[145]/2017	Methylation of specific genes	Tissue, pancreatic islets, whole blood	Unmethylated glucokinase gene is more islet-specific than unmethylated INS DNA
[146]/2018	Genome-wide DNA methylation	Whole blood	Methylation mediates T1D risk at five non-HLA loci mainly by influencing local gene expression.
[147]/2019	Methylation of specific genes	Serum	A higher unmethylated *INS* ratio is associated with IAA levels at the time of T1D diagnosis
[148]/2020	Methylation quantitative trait loci (mQTL) analyses	Peripheral blood	Identification of 10 single nucleotide polymorphism probe pairs significantly related to methylation levels prior to the development of T1D
[149]/2021	Methylation of specific genes	Pancreatic islets	Pro-inflammatorycytokines and T1D genetic risk variants regulate CTSH transcription by differential DNA methylation

## Data Availability

No new data were created or analyzed in this study. All data included in this review have been previously published. If further specific data is needed, it may be provided by the corresponding author upon reasonable request.
